# Acupuncture Point Laterality: Investigation of Acute Effects of Quchi (LI11) in Patients with Hypertension Using Heart Rate Variability

**DOI:** 10.1155/2014/979067

**Published:** 2014-05-12

**Authors:** Gerhard Litscher, Wei-Ping Cheng, Guang-Yu Cheng, Lu Wang, Jian Zhao, Daniela Litscher, Ingrid Gaischek, Zemin Sheng, Haixue Kuang

**Affiliations:** ^1^Research Unit for Complementary and Integrative Laser Medicine, Research Unit of Biomedical Engineering in Anesthesia and Intensive Care Medicine, and TCM Research Center Graz, Medical University of Graz, 8036 Graz, Austria; ^2^Heilongjiang University of Chinese Medicine, Harbin 150040, China; ^3^The First Hospital Affiliated to Heilongjiang University of Chinese Medicine, Harbin 150040, China; ^4^Clinical Medicine of Heilongjiang University of Chinese Medicine, Harbin 150040, China; ^5^Privatklinik Laßnitzhöhe, 8301 Laßnitzhöhe, Austria

## Abstract

Hypertension is one of the major risk factors for cardiovascular disease worldwide. Over 70% of the patients use antihypertensive drugs, so nonpharmacological treatments in addition to the medication are important. Our goal was to investigate acupuncture treatment on the Quchi acupoint using heart rate (HR) and heart rate variability (HRV) and to find out whether there is a laterality in acute effects. Sixty hypertensive patients (36 female, 24 male; mean age ± SD 55.8 ± 9.7 years) were randomly assigned to two manual needle acupuncture groups (group A: left Quchi (LI11) acupoint, group B: right Quchi acupoint). There was a significant (*P* < 0.05) decrease in HR immediately after inserting and stimulating the needle at the left and the right Quchi acupuncture point. In contrast, total HRV increased immediately after inserting the needle, but this increase was significant only towards the end of the stimulation phase and after removing the needle. There were some differences between stimulation of the left and right Quchi acupoint, but they remained insignificant. This study provides evidence that there is a beneficial effect on heart rate variability in patients with hypertension and that there are some effects of laterality of the acupoint Quchi.

## 1. Introduction

Hypertension is known to be one of the major risk factors for cardiovascular disease all over the world. High blood pressure increases the risk for heart disease and stroke, which are the leading causes of death in the United States of America. Hypertension was a primary or contributing cause of death for more than 348,000 Americans in the year 2009, which means about 1000 cases of death each day [1]. Hypertension is also a big problem for the health care systems worldwide. Statistics from the United States of America (2010) show that 31% of American adults (67 million) have high blood pressure [2]. The costs of high blood pressure for the nation are about $47.5 billion each year [3]. In the industrial nations, between 10 and 55% of the whole population suffer from high blood pressure. Leader of this statistic is Germany (55%). In the other European countries, 44% of people have hypertension. The older a person is, the more the probability to have high blood pressure increases. Only one person in ten of 30-year olds suffers from hypertension, but at the age of 50, this number got tripled [4]. The prevalence of hypertension in developed countries is around 40%, whereas in developing countries the prevalence is estimated to be 25%. Also in Asia hypertension becomes more and more a big problem. Statistics show that the overall prevalence for hypertension in the Indian and Chinese population was 22.6% in women and 20.6% in men. Already about a quarter of the population worldwide has high blood pressure. Experts warn that this percentage will increase to about 29% by 2025 [5, 6]. More than 70% of the patients have to use two or more antihypertensive drugs. So a lifestyle modification and other nonpharmacological treatments in addition to the medication are very important for patients with hypertension [7].

The English word “left” comes from the Anglo-Saxon word “lyft,” which means “weak” or “useless,” and the English word “right” is derived from the Anglo-Saxon word “riht,” meaning also “straight” or “correct.” Similarly, in many cultures “right” also means “correct.” In medicine in general and especially in brain research, many studies and reviews deal with the topic laterality of motor and sensory control [8]. However, acupuncture point laterality has not been investigated extensively in the past [9, 10].

The goal of our study was to investigate basic mechanisms of acupuncture treatment on the Quchi acupoint in patients with hypertension using heart rate (HR) and heart rate variability (HRV). Within this investigation we wanted to find out whether there is a laterality in acute effects using needle acupuncture. Previous investigations from China indicated such laterality-related effects [9, 10].

## 2. Subjects and Methods

### 2.1. Patients

At the Heilongjiang University in Harbin, 60 patients (36 female, 24 male; mean age ± SD 55.8 ± 9.7 years; range 34–74 years) suffering from hypertension were investigated. They were randomly assigned to two intervention groups; group A received manual needle acupuncture at the left Quchi (LI11) acupoint and group B received manual needle acupuncture at the right Quchi acupoint. Randomization was performed by chance by a medical doctor who was not involved in this study otherwise. The demographic data of the two groups can be seen in Table 1. All patients were under the influence of blood pressure medication (irbesartan 150 mg oral per day, angiotensin-II receptor antagonist mainly used in the treatment of hypertension). The study was approved by the ethic committee of the Heilongjiang University of Chinese Medicine (number 2010HZYLL-030) and carried out in compliance with the Declaration of Helsinki. All patients gave oral informed consent.

### 2.2. Heart Rate Variability (HRV) and Teleacupuncture

The following quotes are taken from [11].

The duration of RR-intervals is measured during a special time period (5 min), and on spectral analysis basis HRV is determined. Electrocardiographic (ECG) registration is performed using three adhesive electrodes (Skintact Premier F-55; Leonhard Lang GmbH, Innsbruck, Austria) which are applied to the chest.

The researchers in China used a medilog AR12 HRV (Huntleigh Healthcare, Cardiff, United Kingdom) system from the TCM Research Center at the Medical University in Graz for the joint investigations. This system has a sampling rate of 4096 Hz and can therefore detect R-waves extremely accurately. The raw data are stored digitally on a CompactFlash (CF) 32 MB memory card. After removing the card from the portable system, the data were read by a card reader connected with a standard computer in China and then transferred to the TCM Research Center Graz via internet. With a new software the biosignals were analyzed and HRV was displayed in a way to help to judge the function of the autonomic nervous system. Viewing this innovative kind of analysis helps to show how well the human body reacts to sport, stress, recovery, and also acupuncture.

Similar to previous studies, mean HR, total HRV, and the LF (low frequency)/HF (high frequency) ratio of HRV were chosen as evaluation parameters, as such being recommended by the Task Force of the European Society of Cardiology and the North American Society of Pacing and Electrophysiology [12].

### 2.3. Clinical Acupuncture and Procedure

**Figure 1:**
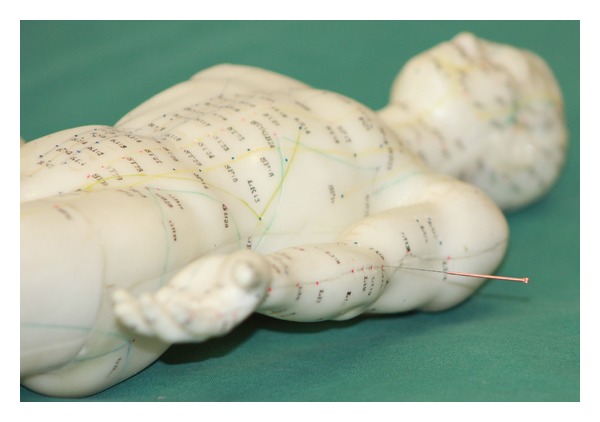
Acupuncture at the left Quchi acupoint (LI11).

**Figure 2:**
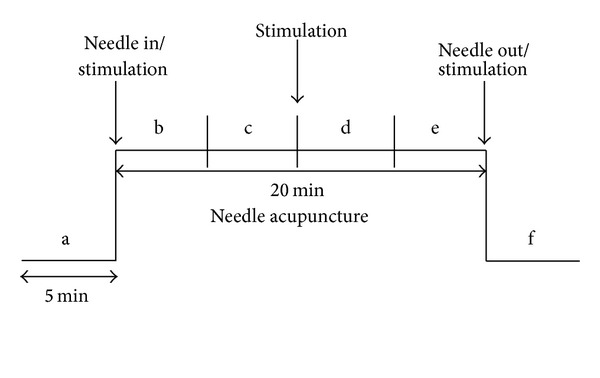
Measurement procedure. The data before (measurement phase a), during (b–e), and after (f) manual needle acupuncture stimulation at the Quchi acupoint (compare Figure 1) were measured and statistically analyzed.

All patients received manual needle acupuncture at the Quchi acupoint (LI11) on the arm (Figure 1). With the elbow flexed at a right angle, Quchi is located at the lateral end of the cubital crease and at the midpoint connecting the radial end of the transverse cubital crease and the external humeral epicondyle [13]. Sterile single-use needles (0.25 × 25 mm; Huan Qiu, Suzhou, China) were used. Needling was performed perpendicularly (depth 1–1.5 cun), and the needle was stimulated clockwise and counterclockwise for 15 seconds each, with six rotations per second, resulting in 90 rotations per stimulation. Stimulation was done immediately after inserting the needle, 10 minutes later, and before removing the needle (compare Figure 2).

### 2.4. Statistical Analysis

The data were analyzed using SigmaPlot 12.0 software (Systat Software, Chicago, USA). Graphical presentation of results uses box plot illustrations. Testing was performed with repeated measures ANOVA on ranks and Tukey test. The criterion for significance was *P* < 0.05.

## 3. Results

**Figure 3:**
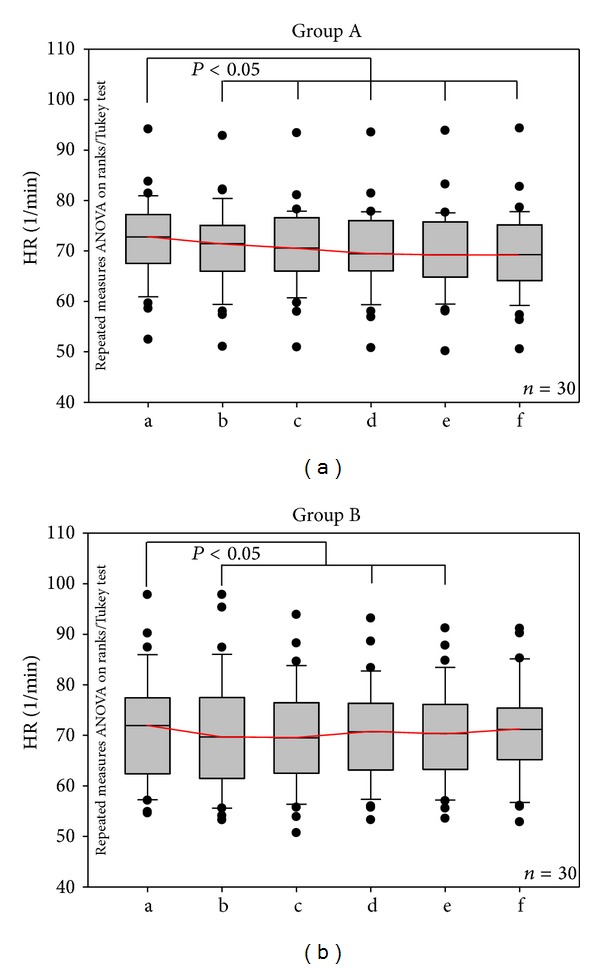
Mean heart rate in group A (Quchi left) and group B (Quchi right). Box plot illustration of changes before (a), during (b–e), and after (f) needle acupuncture. Significant changes were found in both groups. The horizontal line in the box gives the position of the median. The end of the box defines the 25th and 75th percentile; the error bars mark the 10th and 90th percentile.

The results of mean HR from the ECG recordings before, during, and after acupuncture of the 60 patients with hypertension are shown in Figure 3. There was a significant (*P* < 0.05) decrease in HR immediately after inserting and stimulating the needle at the left (group A) and the right (group B) Quchi acupuncture point. HR remained on that level during the whole experiment.

**Figure 4:**
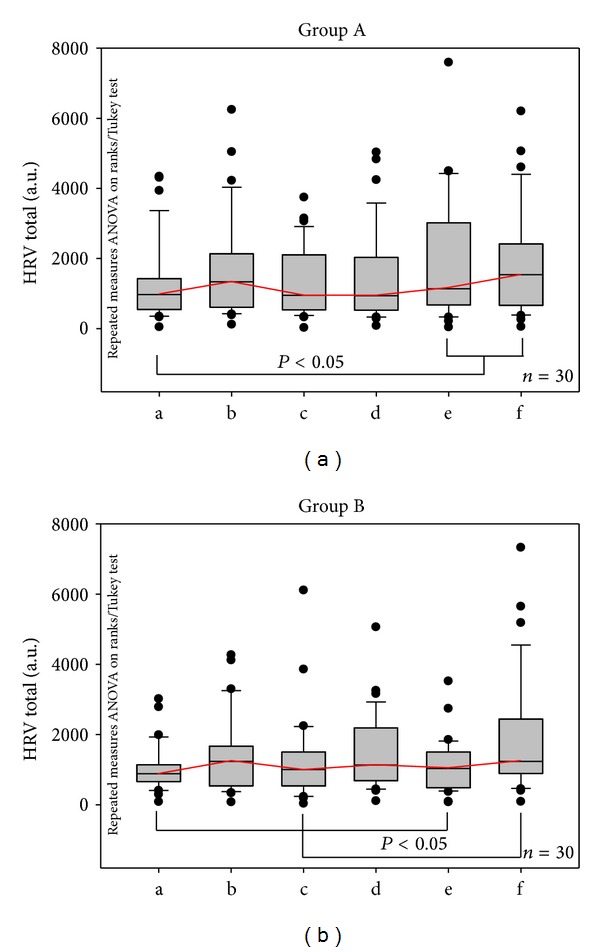
Total heart rate variability. Graphical box plot presentation of significant changes at the end of the acupuncture session. Note the increase in total HRV after each manual needle stimulation (b, d, and f). For further explanations compare Figures 2 and 3.

In contrast to this decrease in HR, total HRV increased immediately after inserting the needle (Figure 4). However, this increase did not reach the level of significance. Only towards the end of the stimulation phase and also after removing the needle, the increase in total HRV was significant (*P* < 0.05). There were some differences between stimulation of the left and right Quchi acupoint, but they remained insignificant.

**Figure 5:**
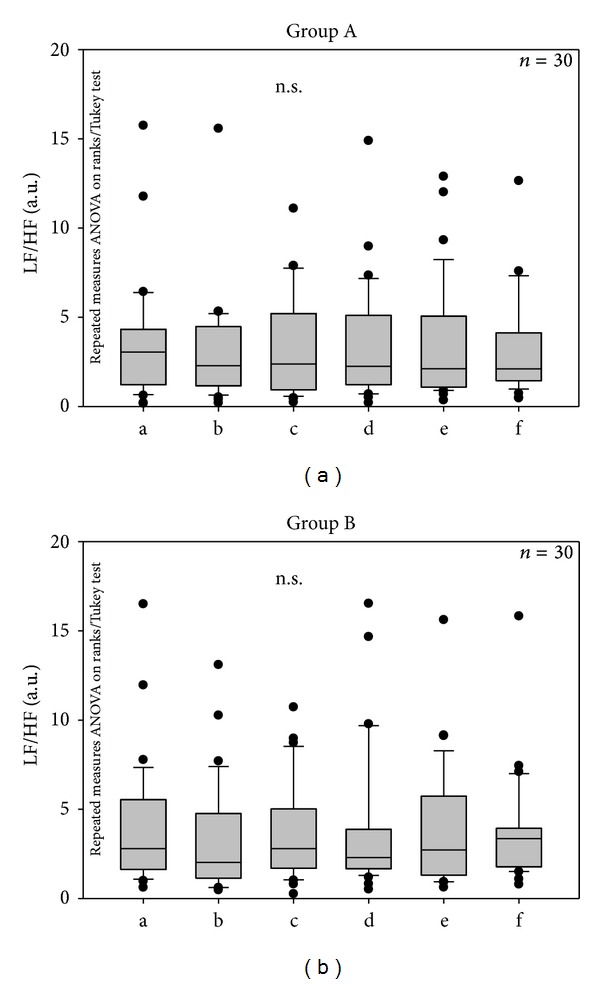
LF (low frequency)/HF (high frequency) ratio during acupuncture treatment in the 30 patients of each group. For further explanations see Figure 3.

No significant changes could be seen in the LF/HF ratio during acupuncture (see Figure 5).

There was no significant change in systolic or diastolic blood pressure (BPsys and BPdia) before and after acupuncture.

## 4. Discussion

Laterality in acute effects after needle acupuncture stimulation has not been studied extensively in the past. One pilot study on the topic, which is also based on evidence from HRV like our present study, was published by Wang et al. last year [9]. The authors investigated the Neiguan acupoint (PC6) and whether it has any laterality. Eighteen healthy female volunteers were included in this pilot study. The results from the research team at the China Academy of Chinese Medical Sciences, Beijing, showed that there were significant differences in the standard deviation of RR intervals and also in the HRV total power between the left PC6 stimulation group and the right PC6 stimulation group. The authors concluded that PC6 may have laterality, but they also stated that only PC6 was investigated and so they cannot be sure whether all acupoints have laterality and whether this laterality will change under different conditions, such as different disorders or aging [9].

HRV can be used as a globally reliable indicator of the state of health. It could be demonstrated in previous investigations that in special syndromes like stress or burnout one can counteract this process using different preventive methods like acupuncture [14].

The results of our present investigation also did not show significant differences in laterality with regard to HR; however, similar to the pilot study by Wang et al. [9], there was a trend towards a higher level in total HRV after stimulation of the left Quchi acupoint, but the values did not differ significantly. The number of patients in each group was *n* = 30, so that the comparison of the two groups (A and B) is representative. Anyway, similar to previous investigations, we are not sure at the moment whether this phenomenon can change under different brain activities because we did not measure other specific brain neuromonitoring parameters concerning central nervous system regulation.

The same acupoint (Quchi) was also used to investigate the mechanism of acupuncture on blood pressure and blood plasma catecholamines in patients with essential hypertension [15]. Therefore, similar to our present study, 60 patients were investigated. Thirty patients received electroacupuncture on Quchi (bilateral); the control group (*n* = 30) received western medicine (nicardipine). The effective rate 66.7% in the electroacupuncture group was similar to that of 70.0% in the medication group. The authors concluded that also electroacupuncture on Quchi is able to beneficially regulate blood pressure of patients with essential hypertension through adjusting blood plasma catecholamines [15].

A further article on the topic deals with an animal experimental study [16]. Quchi and other acupoints were stimulated electrically in 60 spontaneous hypertension rats. The authors from China concluded that acupuncture of Quchi can effectively lower both systolic and diastolic blood pressure [16].

An effect of acupuncture at Quchi and also at Taichong (LR3) on blood pressure was observed not only in older patients, but also in younger patients with hypertension [17]. Again, 60 hypertensive persons were randomized into an electroacupuncture group and a western medication group (captopril), 30 patients in each group. Acupuncture on Quchi reduced systolic and diastolic blood pressure, and there was no statistically significant difference between the two groups. This study showed that stimulation of the acupoint Quchi has long-term antihypertensive effects and effectively improves day-night rhythm in young subjects with hypertension [17].

In our present study, no side effects appeared. We had no elbow soreness, as described in other articles [18]. Every enrolled patient tolerated the procedure and finished the study.

As mentioned in the introduction, lifestyle is associated with HRV and blood pressure [19, 20]. However, there are only few studies on HRV condition and short-term, stimulation-based changes in hypertensive patients. The results concerning the possible lateralization are consistent with previous findings for Asian populations and confirm these results [9]. Further studies in Europe and in America are necessary because, in the Asian population, individuals have much lower body mass indices [21–23].

In conclusion, this study provides evidence that there is a beneficial effect on heart rate variability in patients with hypertension and that there are some effects of laterality of the acupoint Quchi, although these effects did not reach the level of significance. The question of laterality of acupoints still needs further investigations.

## Figures and Tables

**Table 1:** Demographic data of the two study groups. Data are given as mean ± SD.

	Group A	Group B	A + B
Number of patients	30	30	60
Age (years)	55.7 ± 10.2	55.9 ± 9.4	55.8 ± 9.7
Sex	23 f, 7 m	13 f, 17 m	36 f, 24 m
BPsys (mmHg)	152.3 ± 13.9	143.2 ± 11.1	147.8 ± 12.6
BPdia (mmHg)	94.8 ± 6.8	88.6 ± 8.3	91.7 ± 7.7
